# Variations in morphology and PSII photosynthetic capabilities during the early development of tetraspores of *Gracilaria vermiculophylla *(Ohmi) Papenfuss (Gracilariales, Rhodophyta)

**DOI:** 10.1186/1471-213X-10-43

**Published:** 2010-04-28

**Authors:** Xiujun Xie, Guangce Wang, Guanghua Pan, Shan Gao, Pu Xu, Jianyi Zhu

**Affiliations:** 1 Key Laboratory of Marine Resources and Chemistry, College of Marine Science and Engineering, Tianjin University of Science and Technology, Tianjin 300457, China; 2 Institute of Oceanology, Chinese Academy of Science, Qingdao 266071, China; 3 Department of Biology, Changshu Institute of Technology, Changshu 215500, China

## Abstract

**Background:**

Red algae are primitive photosynthetic eukaryotes, whose spores are ideal subjects for studies of photosynthesis and development. Although the development of red alga spores has received considerable research attention, few studies have focused on the detailed morphological and photosynthetic changes that occur during the early development of tetraspores of *Gracilaria vermiculophylla *(Ohmi) Papenfuss (Gracilariales, Rhodophyta). Herein, we documented these changes in this species of red algae.

**Results:**

In the tetraspores, we observed two types of division, cruciate and zonate, and both could develop into multicellular bodies (disks). During the first 84 hours, tetraspores divided several times, but the diameter of the disks changed very little; thereafter, the diameter increased significantly. Scanning electron microscopy observations and analysis of histological sections revealed that the natural shape of the disk remains tapered over time, and the erect frond grows from the central protrusion of the disk. Cultivation of tissue from excised disks demonstrated that the central protrusion of the disk is essential for initiation of the erect frond. Photosynthetic (i.e., PSII) activities were measured using chlorophyll fluorescence analysis. The results indicated that freshly released tetraspores retained limited PSII photosynthetic capabilities; when the tetraspores attached to a substrate, those capabilities increased significantly. In the disk, the PSII activity of both marginal and central cells was similar, although some degree of morphological polarity was present; the PSII photosynthetic capabilities in young germling exhibited an apico-basal gradient.

**Conclusions:**

Attachment of tetraspores to a substrate significantly enhanced their PSII photosynthetic capabilities, and triggered further development. The central protrusion of the disk is the growth point, may have transfer of nutritive material with the marginal cells. Within the young germling, the hetero-distribution of PSII photosynthetic capabilities might be due to the differences in cell functions.

## Background

Red algae are primitive photosynthetic eukaryotes that are widely distributed around the world, and they are ideal subjects for studies of photosynthesis and development. Red algae strongly resemble to cyanobacteria: Both are characterised by the presence of phycobilisomes, which consist of phycobiliproteins, which function as a photosynthetic antenna system with chlorophyll a [1]. Red algae exhibit alternation of generations. The spores, which are produced from sporophytes, are large spherical cells that can develop rapidly into mature individuals under laboratory conditions. Furthermore, the release of spores can be controlled easily by photoperiod [2,3].

Guiry [4] previously described the germination patterns of red algae spores as consisting of five representative types: *Nemalion, Gelidium, Naccaria, Ceramium, and Dumontia*. *Nemalion*-type and *Gelidium*-type germination are very similar to each other and, are less common among the red algae than the other types [5]. The *Naccaria*-type is common in Bonnemaisoniales and in many of the primitive members of the Gigartinales [6]. The *Ceramium-type*, which exhibits a bipolar germination pattern, is found in Bangiophycidae and Ceramiales [7-9]. The *Dumontia*-type generally occurs in advanced members of Gigartinales, Rhodymeniales and Gracilariales [6,10-13]. Of the five representative types, the first four share some common features. After attachment to a substrate, the spore first forms one or more protuberance(s), from which the apical cell and a rhizoid are produced. In the *Dumontia *type of germination, the spore develops into a mass of small cells within the original spore wall, and that multicellular body then grows into a disk that stems from the division of marginal cells of the mass. Using scanning electron microscopy (SEM), Chen and Taylor (1976) investigated *Dumontia*-type development in *Condrus crispus *and concluded that the extracellular sheath is very important for its development [12]. Thus, this type of germination differs from the other four types.

Species of *Gracilaria*, whose spores exhibit *Dumontia*-type germination, are economically important algae, and thus they have received a lot of attention from researchers [14-17]. The *polysiphonia*-type life history of *Gracilaria *consists of two isomorphic generations (diploid tetrasporophyte and haploid gametophyte) [18-20], as well as a parasitic heteromorphic carposporophyte generation. Tetraspores released from the tetrasporophyte play a crucial role in the life cycle of *Gracilaria *and have been investigated extensively [19,21,22]. Although spore germination has been studied intensively [11,23,24], few studies have focused on the detailed morphological and photosynthetic changes at the single-cell level that occur during the early development of *Gracilaria *tetraspores. In this study, we investigated the early developmental properties of tetraspores of *Gracilaria vermiculophylla *(Ohmi) Papenfuss, including morphological changes and changes in the photosynthetic capabilities of PSII.

## Results and Discussion

### Two types of division in *G. vermiculophylla *tetraspores

**Figure 1:**
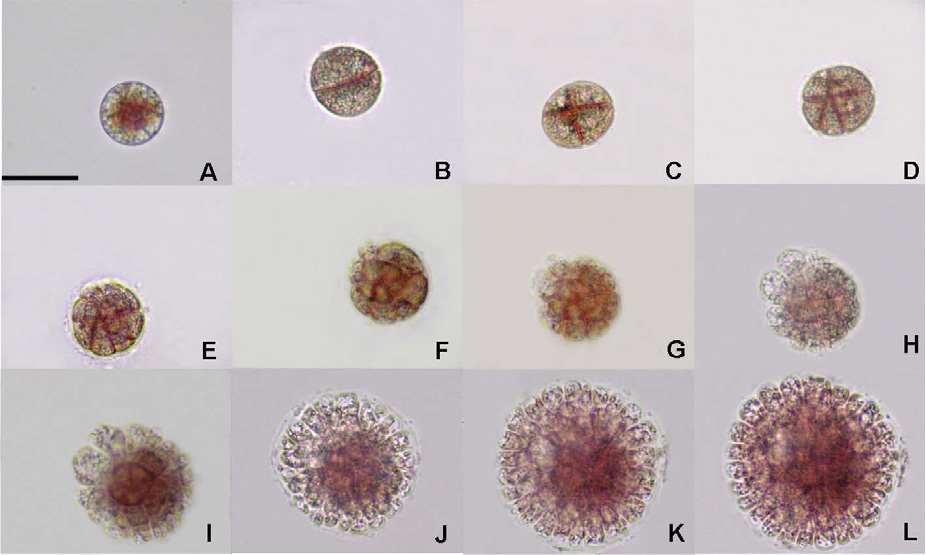
**Cruciate development of a *G. vermiculophylla *tetraspore**. This is the main type of division (98.4% ± 0.0045), and it is characterized by the crossing of the first and second cleavage furrow. A, Freshly released tetraspores; B-H, Various developmental phases at 12 hour intervals; I-L, Various developmental phases at 24 hours intervals. Scale bar = 30 μm.

**Figure 2:**
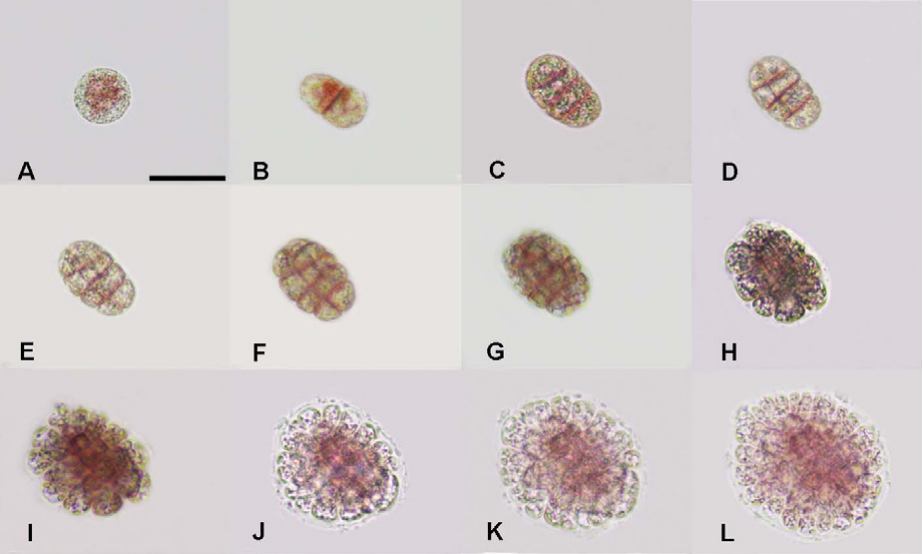
**Zonate development of a *G. vermiculophylla *tetraspore**. This type of division is rarely observed (1.6% ± 0.0045). The spore elongates before the first division, and the second furrow is always parallel to the first one. A, Freshly released tetraspores; B-H, Various developmental phases at 12 hour intervals; I-L, Various development phases at 24 hour intervals. Scale bar = 30 μm.

Freshly released tetraspores of *G. vermiculophylla *were spherical, with a diameter of about 26 ± 0.05 μm (Figures 1A and 2A). Eight hours after release, most of the tetraspores had adhered to the substrate and began to divide. As is shown in Figures 1 and 2, two division types were observed: the cruciate type accounted for approximately 98.4% ± 0.0045, and the zonate type accounted for about 1.6% ± 0.0045. These two division types differ from each other mainly in the first and second divisions. In the cruciate type, spherical tetraspores divide into two equal parts by a cleavage furrow, which is always located at the equator of the tetraspore (Figure 1B); the second cleavage furrow is always perpendicular to the first one, leading to the formation of cruciate tetraspores (Figure 1C). In the zonate type, the spore elongates before the formation of the first median division furrow (Figure 2B), and the second furrow parallels the first one (Figure 2C). Thereafter, all of the spores developed into a hemispherical multicellular body, called the disk. After cultivation for 84 hours, the central part of the disk grew upright, while the marginal part grew centrifugally, which resulted in a rapid increase in the diameter of the disk (Figure 1F-J; Figure 2G-J).

### Changes in diameters of tetraspores and disks

**Figure 3:**
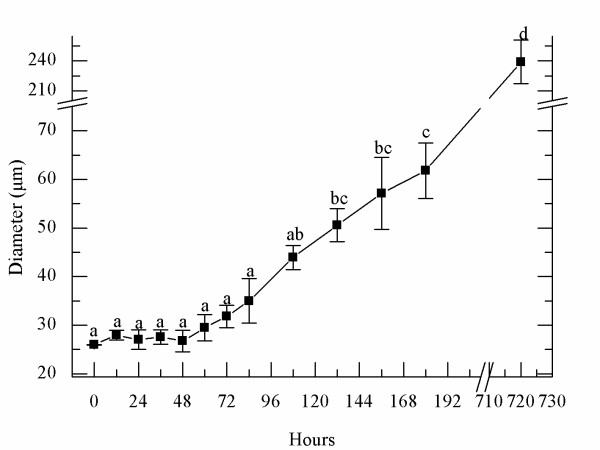
**Variations in tetraspore/disk diameter during cruciate development**. The time point at which the spores were freshly released was set as 0 hours. Statistical results suggest that there is no significant difference in diameter of spores or disks before 84 hours. After 84 hours, morphological changes occurred in marginal cells of the disk, the diameter began to increase (refer to Figure1-A-H). A one-way ANOVA and Students-Newman-Keuls Post Hoc comparison were used to test for statistical significance.

Throughout the developmental process, changes in the diameter of spores were uneven. At 12-hour intervals during the first 84 hours, the mean diameter of spores was 26 ± 0.05 μm, 28 ± 1.0 μm, 27 ± 1.5 μm, 27 ± 2.2 μm, 29 ± 2.7 μm, 32 ± 2.3 μm, and 35 ± 4.6 μm, respectively (Figure 3). Although the spores divided several times during this time interval, their volume changed little (P > 0.05). After cultivation for 84 hours, the diameter of disks increased dramatically, from 43.9 ± 2.5 μm at 108 hours to 50.6 ± 3.4 μm at 132 hours (P < 0.05). After cultivation for 720 hours (30 days), the diameter was about 240 ± 21.9 μm, and at this time the erect frond had been visible for 7-8 days.

The development pattern of the tetraspores of *G. vermiculophylla*, along with other species of *Gracilaria *[19], falls within the framework of the *Dumontia*-type germination described above. This scenario differs significantly from the development of conchospores in *Porphyra *(Rhodophyta) described by Fan *et al. *(2008), in which the conchospores elongate when the first cell division occurs. With further divisions of conchospores, the longitudinal length of the conchospores increased [25].

### Changes in PSII photosynthetic capabilities of tetraspores and disks during development

**Figure 4:**
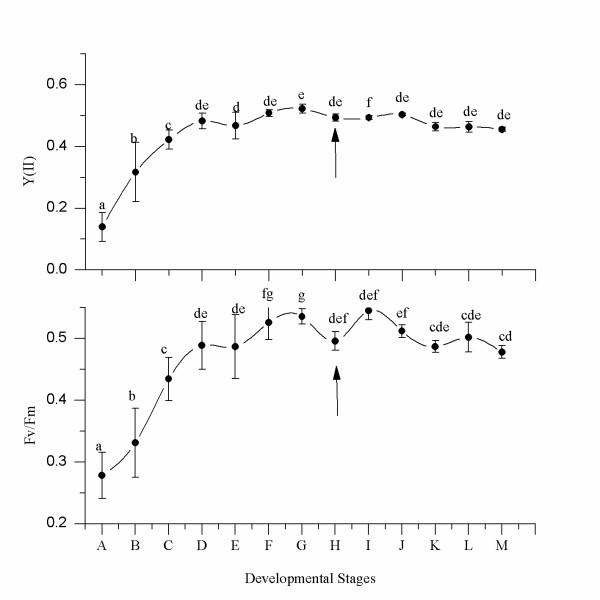
**Changes in Y (II) and Fv/Fm during early development of *G. vermiculophylla***. A, In freshly released tetraspores, both Fv/Fm and Y (II) were very low; B, The PSII photosynthetic capabilities in attached tetraspores before the first division, compared with A, were enhanced significantly; C, Attached tetraspores after the first division; D, Attached tetraspores after the second division. There was no significant difference in the photosynthetic capabilities of PSII among E-M; E-M, multicellular bodies (disks) at different developmental stages. Statistical significance was tested by one-way ANOVA and Student-Newman-Keuls post hoc comparison. The arrow shows the start of tissue cultivation of excised disk. Y (II) was determined under constant illumination of 156 μmol photon m^-2^.s^-1^.

Two important photosynthetic properties of PSII, maximal PSII quantum yield (Fv/Fm) and effective PSII quantum yield (Y (II)), were measured during early development of tetraspores of *G. vermiculophylla*. Figure 4 illustrates the changes in Fv/Fm and Y (II) with developmental stage. The photosynthetic capabilities of the suspended tetraspores freshly released from the tetrasporophytes were quite limited, and the values of Y (II) and Fv/Fm were about 0.14 ± 0.046 and 0.28 ± 0.04, respectively. However, 8-10 hours later these values for the attached tetraspores increased dramatically to 0.32 ± 0.097 and 0.33 ± 0.06, respectively. After the first division of the attached tetraspores, the values of Y (II) and Fv/Fm reached 0.42 ± 0.03 and 0.43 ± 0.04, respectively. The increase in photosynthetic competence continued until the tetraspores developed into the quartered stage, when the values of Y (II) and Fv/Fm were 0.48 ± 0.025 and 0.49 ± 0.04, respectively. Thereafter, the values of Fv/Fm and Y (II) remained stable. Statistical analysis revealed significant differences in the values of Y (II) and Fv/Fm during the first four stages (i.e., suspended, attachment, dimidiate, and quartered) (P < 0.05), and no significant differences in the following developmental stages (P > 0.05).

In freshly released tetraspores that were in suspension for about 8 hours, both Fv/Fm and Y (II) remained very low, suggesting that they were dormant and maintained only suspended metabolism. After attachment, their photosynthetic capabilities increased dramatically (Figure 4) and were accompanied by rapid division. Thus, the attachment of tetraspores enhanced their photosynthetic capabilities of PSII. In addition, only attached tetraspores began to divide, whereas none of the suspended tetraspores did. Therefore, attachment must play a great role in triggering further development. The promoting effect of attachment on further development was reported for the conchospores of *Porphyra *(Rhodophyta)[25], as well, because only attached conchospores could develop normally. Similar results have been reported for algae, such as some species of Rhodophyta [26,27], Phaeophyta [28], and Chlorophyta [29]. Thus, it seems that attachment is essential for spores to develop.

### Initiation of the erect frond

**Figure 5:**
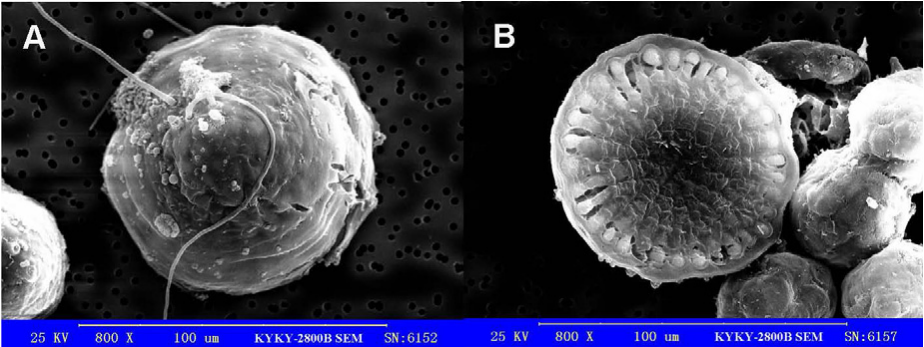
**SEM micrographs of disks**. The disks were cultured for 13 days after the attachment of tetraspores and then were detached from the substrate for the SEM observations. A and B were observed from different angles.

**Figure 6:**
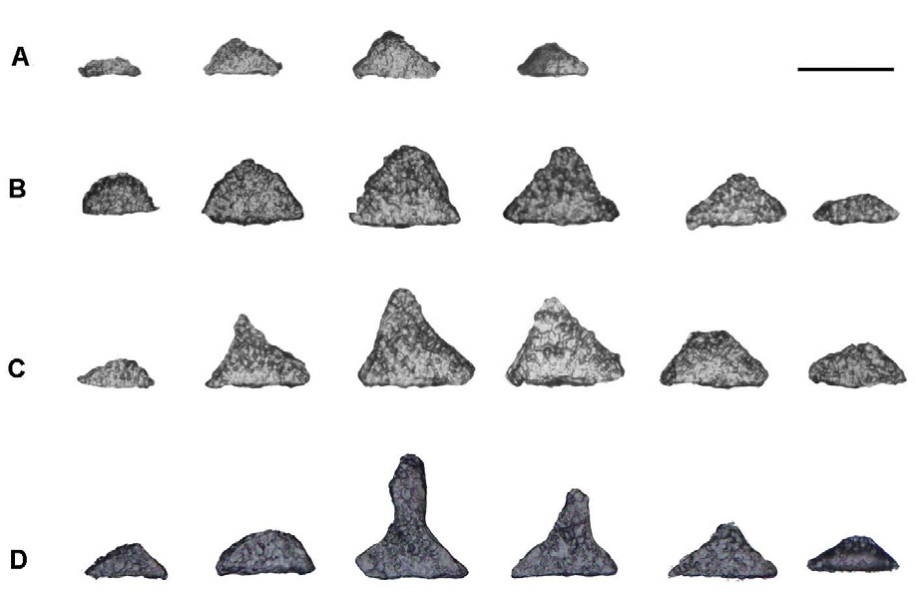
**Photos of serial paraffin slices of disks at various developmental stages**. Disks cultured 14, 15, 16, and 20 days after the attachment of tetraspores were detached from the substrate for preparation of serial paraffin slices. A, After cultivation for 14 days; B, After cultivation for 15 days; C, After cultivation for 16 days; D, After cultivation for 21 days. Scale bar = 100 μm.

**Figure 7:**
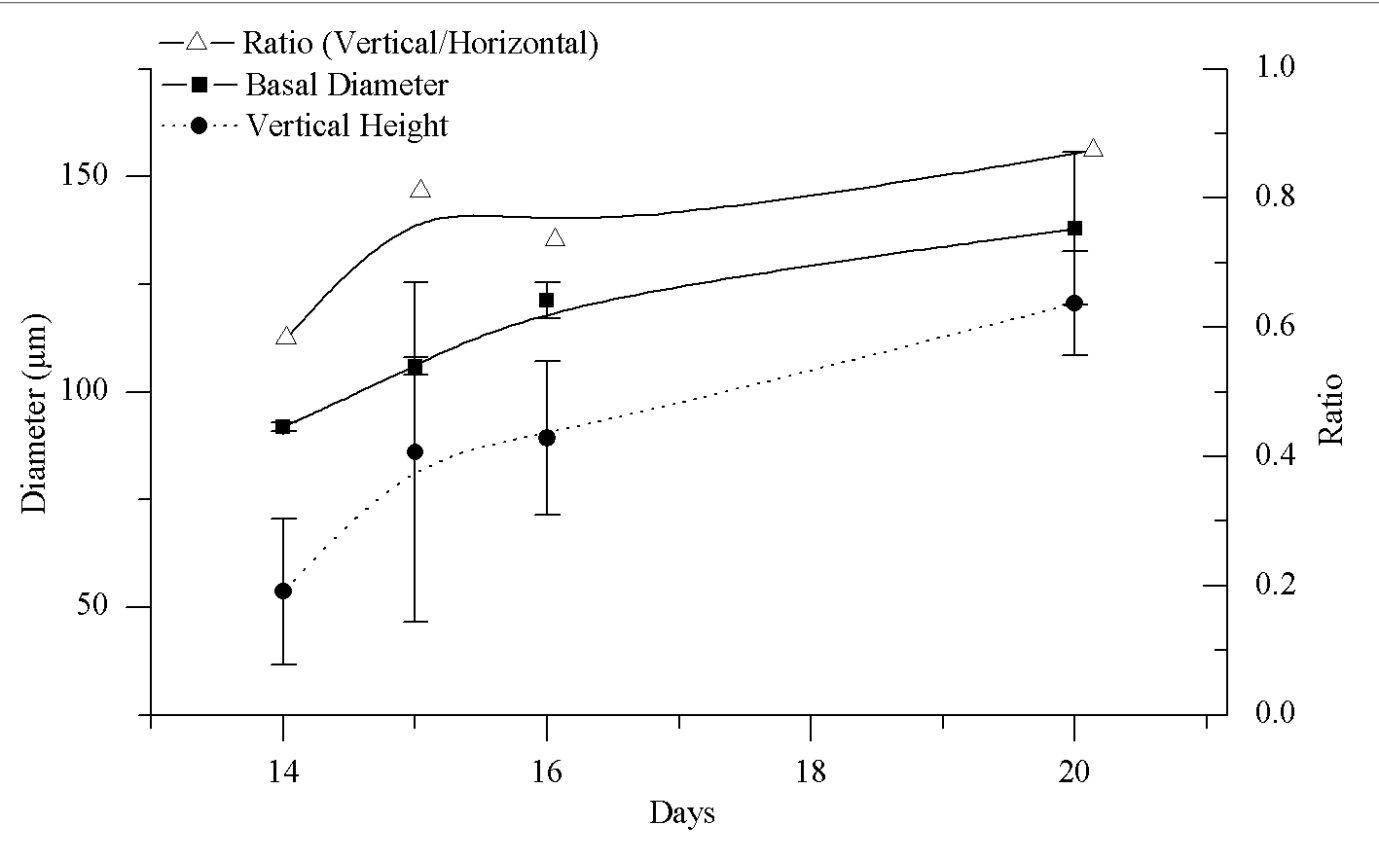
**Comparison of basal diameter and vertical height of disks at various developmental stages**. The basal diameter (solid black square) and vertical height (solid black circle) were measured in photos of serial paraffin slices, and the ratio was calculated (hollow triangle).

**Figure 8:**
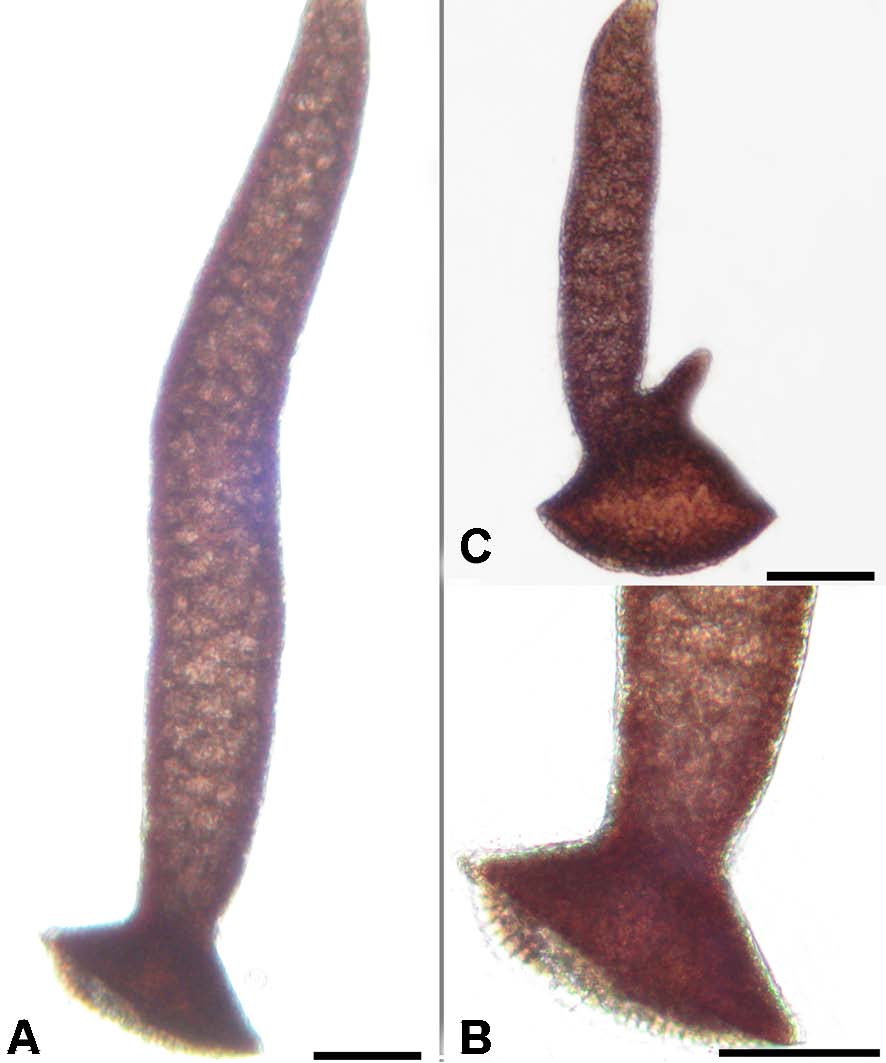
**Germlings of *G. vermiculophylla *cultured for 30 days showing the erect axis**. A, A young germling with an erect frond detached from the substrate; B, A close-up view of A; C, a young germling with two erect fronds. Scale bars = 100 μm.

The division of marginal cells leads to an increase in basal diameter, whereas the division of central cells results in the formation of the protrusion (i.e., the erect frond). SEM photographs revealed the tapered shape of the disk (Figure 5), which also was demonstrated in the photos taken of the serial paraffin slices. As seen in Figures 6 and 7, after cultivation for 14 days the disk section was tapered and the basal diameter and vertical height were about 92.0 ± 1 μm and 53.7 ± 16.9 μm, respectively (Figures 6A and 7). By day 15 these values were 106 ± 2 μm and 86 ± 39.4 μm, respectively (Figures 6B and 7), and at day 16 they were 121.3 ± 4.2 μm and 89.3 ± 17.9 μm, respectively (Figures 6C and 7). By day 21, an erect frond had been initiated from the center of the disk, and the horizontal and vertical lengths of the frond were 138 ± 17.8 μm and 120.7 ± 12.1 μm, respectively (Figures 6D and 7). During this period (from 14 to 21 days), the rate of increase of the vertical height of disks was greater than that of the basal diameter, based on a comparison of the ratio of vertical height to basal diameter (Figure 7). A month later, the sporelings could reach 2 mm in length (Figure 8).

**Figure 9:**
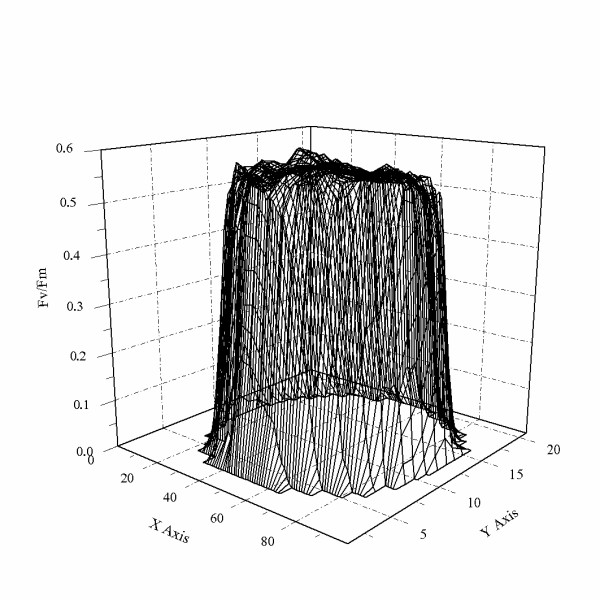
**The 3D profile of the maximal PSII quantum yield (Fv/Fm) of a disk**. Using the transect tool in the Imaging Win v2.21d software, the distribution of Fv/Fm within a disk, which was cultured for 13 days after the attachment of the tetraspore, was analyzed. It is clear that PSII photosynthetic capabilities of different areas within the disk are very similar. The Z-coordinate represents the value of Fv/Fm.

The transect tool in the ImagingWin v2.21d software was used to analyze the spatial heterogeneity of photosynthetic properties across disks that were cultivated 13 days after attachment and were about 100 μm in diameter. Figure 9 shows the 3-D profile of the value of Fv/Fm for a disk. Little fluctuation in the value of Fv/Fm was found, and most parts of the disk had a value of ~0.5. The trend for Y (II) was similar (not shown).

These results demonstrate that the central protrusion of the disk plays a crucial role in the disk stage of development of tetraspores; it is the growth point of the disk and it controls the initiation of the erect frond. In contrast, the marginal cells of the disk grow centrifugally and always attach to the substrate. It is noteworthy that a degree of morphological polarity is present in the marginal cells and the central cell. However, this polarity is not reflected in the photosynthetic capabilities of PSII according to our results (Figure 9). Thus, we believe that the marginal cells not only function as a holdfast to keep the thalli stable in the onrushing tide, which was described previously by Li [30], but that they also support the growth of the central protrusion by transition photosynthate, since the pit connection, from which nutrients are transferred from hosts cells to their parasites [31,32], is generally occurred among the cells of disk in some species in the genus *Gracilaria *[33].

### Repair of the excised disk

**Figure 10:**
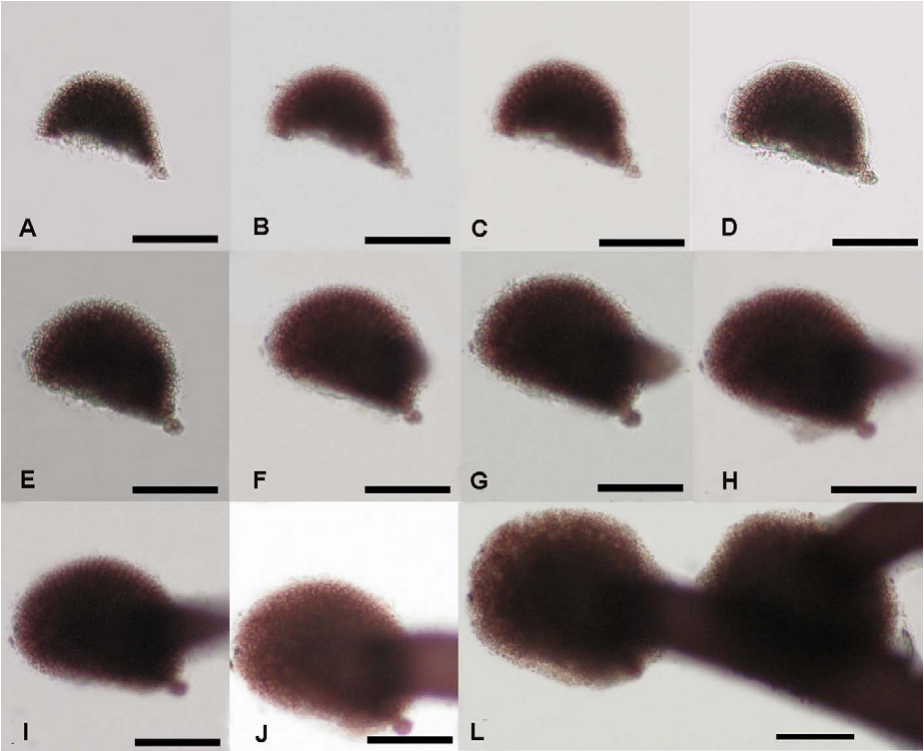
**The repair process of excised disks that contained the protrusion**. Disks cultured for 13 days were excised in situ, and then the remaining part with the central protrusion was cultivated continuously. A-J, At 24 hours intervals after cutting. K, 2 weeks after cutting. Scale bars = 100 μm.

**Figure 11:**
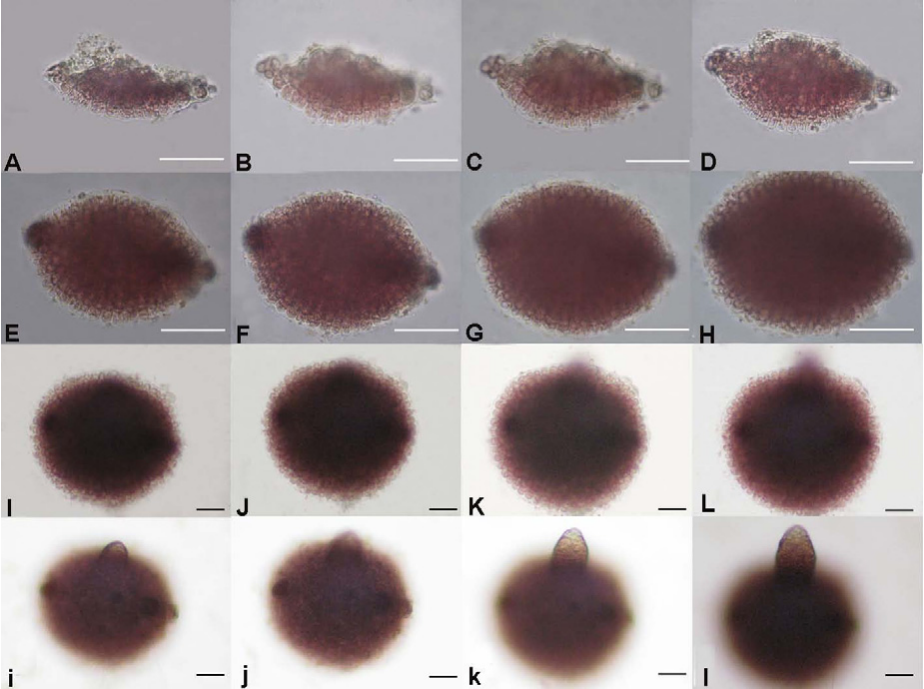
**The repair process of excised disks that did not contain the protrusion**. Disks cultured for 13 days were excised in situ, and then the remaining part without the central protrusion was cultivated continuously. They formed a spindle-like disk (A-H) first and then initiated the erect frond at the center of the new disk (I, i). A-L, At 24 hours intervals; I and i, J and j, K and k, and L and l, were captured at the same time but with different focus, respectively. Scale bars = 50 μm.

Disks that had been cultured for 13 days were excised in situ and then cultivated, and the remaining parts that were attached to the substrate also continued to be cultivated. As is shown in Figures 10 and 11, we used two different cutting proportions: In the first, the margin of the disk was cut off and a semicircular part accounting for ~60% of the whole disk remained (Figure 10A), in the second most of the disk, including the protrusion, was cut off, leaving only the margin (about 20% of the whole disk) of the disk (Figure 11A). The growth direction of the residual parts of the two groups differed. In the first case, the remaining part developed normally after cutting, and repair of the wound was very slow (Figure 10B-E). Six days after cutting, the erect frond was initiated from the center of the disk (Figure 10F-J), which was semicircular in shape until 2 weeks later, when the semicircular disk had developed into a sporeling with a circular holdfast and an erect axis 500 μm in length (Figure 10K). In the second case, the division of the marginal intact cells of the remaining part ceased until a new symmetrical spindle-like disk formed; this likely was due to the rapid division of cells at the wound side. Before the formation of the new spindle-like disk, its longitudinal diameter remained constant (Figure 11B-E). After the spindle-like disk formed, the basal diameter began to enlarge (Figure 11F-H). Nine days after the cutting, the spindle-like disk had become a circle, and the erect frond began to initiate from the center of the disk (Figure 11I, i-L, l). In comparison with the first case, the initiation of the erect frond in the second case was delayed by ~3 days (Figure 10F and Figure 11I, i).

**Figure 12:**
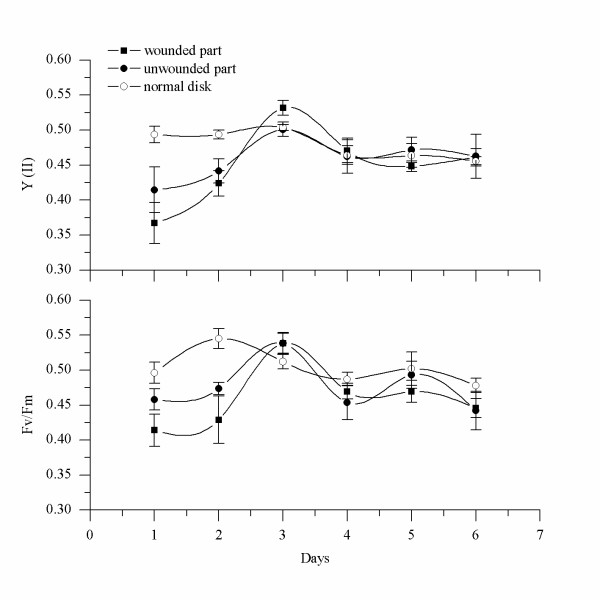
**Changes in photosynthetic capabilities of excised disks that contained the protrusion**. The photosynthetic capabilities of PSII in excised disks were determined every day to compare them with the abilities of normal disks. Y (II) was determined under constant illumination of 156 μmol photon m^-2^.s^-1^.

**Figure 13:**
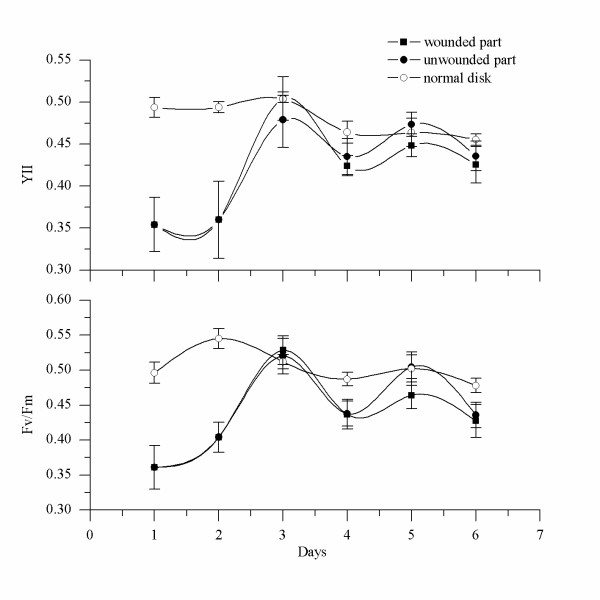
**Changes in photosynthetic capabilities of excised disks that did not contain the protrusion**. Y (II) was determined under constant illumination of 156 μmol photon m^-2^.s^-1^.

Compared to the normal disk, the photosynthetic competence of the excised disk changed considerably during the first 2 days after excision. The values of Fv/Fm and Y (II) for a normal disk were about 0.5, whereas the value for the excised disk during the first 2 days was slightly lower. Figures 12 and 13 illustrate that differences also existed between the groups with different cutting modes. In the case in which the remaining part contained the protrusion (Figure 12), the values of Fv/Fm and Y (II) decreased to 0.4-0.45 and 0.37-0.41, respectively, by the first day after cutting, and these values were dramatically lower than those of the normal disk (P < 0.05). Two days later, however, the values recovered to normal levels. In the case in which the remaining part did not contain the protrusion (Figure 13), after cutting the values of Fv/Fm and Y (II) decreased to 0.36 and 0.35, which were significantly lower than those of the normal disk (P < 0.05). By the third day after excision, these values also had recovered.

These results together with the results of both SEM and analysis of serial paraffin slices validate the premise that the central protrusion of the disk is responsible for the initiation of the erect frond. These data also illustrate the potential regeneration capabilities of the disk. Li (1999) [30] described this phenomenon was described in *Gracilariopsis lemaneiformis*, they found that suspended small pieces cut from the disk could grow into a spherical mass of cells and then initiate many fresh erect fronds around it. In our study, the excised disk remained attached and bore some degree of polarity; thus the initiation of the erect frond always occurred from the central protrusion of the new disk (Figure 11). However, the disk has enormous potential for regeneration, so it might be feasible to take the advantage of this capability for propagation in farming.

### Heterogeneous distribution of photosynthetic capabilities along the length of the young germling

**Figure 14:**
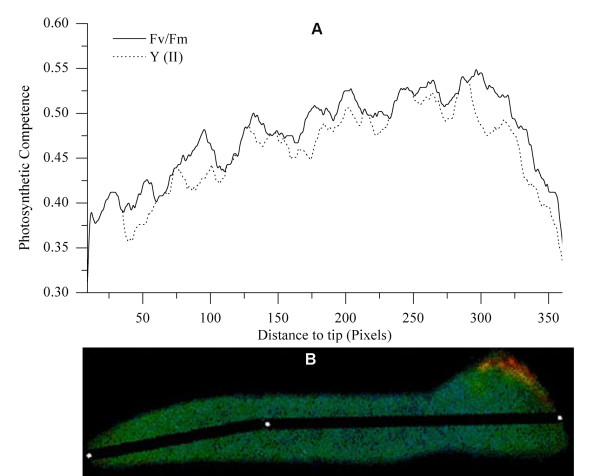
**Heterogeneous distribution of Fv/Fm and Y (II) along the length of a young germling**. The germling was cultured for 30 days after the attachment of the tetraspore. In some parts, the value of Fv/Fm overlaps with that of Y (II). A, PSII Photosynthetic capabilities of different positions within the germling. The values of Fv/Fm and Y (II) represent the mean of the pixel value included in the black line across the young germling (see B); B, False color image of Fv/Fm of the young germling.

Longitudinal transects of young germlings also were analyzed using the transect profile tool. Figure 14 shows that the distribution of Fv/Fm and Y (II) was heterogeneous. The values of Fv/Fm (0.50 ± 0.01) and Y (II) (0.48 ± 0.01) were elevated at the point from which the erect frond initiated. In the distal regions, the values of Fv/Fm and Y (II) were about 0.37 ± 0.02 and 0.37 ± 0.02, which clearly were lower than those from where the erect frond originated (P < 0.05). The values of Fv/Fm and Y (II) for the disk also were lower than that for the root of the frond.

This phenomenon of heterogeneous distribution of photosynthetic ability could be attributed to differences in cell functions within the young germling. *Gracilaria *has uniaxial construction, and the manner of growth is by the division of the apical cell. The daughter cells divided from the apical cell and then elongate and enlarge [34], forming an apico-basal gradient in cell age and cell volume. This gradient corresponds to the distribution of the photosynthetic activities of PSII. The rapid consecutive division of apical cells may result in the incomplete assembly of PSII, which could possibly explain the low photosynthetic activities of apical cells (Figure 14). However, after division, the daughter cells would in turn expand through the disposition of large amounts of wall material, which could be synthesized by high efficient photosynthesis (i.e. high value of Fv/Fm and Y (II)). Meanwhile, the occurrence of the hetero-distribution of PSII photosynthetic capabilities and the pit connections between the thallus cells of *Gracilaria *[33], imply that nutritive materials are transferred from basal cells to apical cells to promote their rapid division.

## Conclusions

The PSII activities of tetraspores increased significantly after the tetraspores attached to the substrate. Attachment is likely triggered their rapid division, and consequently further development was initiated. Through one of two division types, (cruciate or zonate type), attached tetraspores could developed into tapered disk, whose central protrusion was the growth point that controlled the initiation of the erect frond. Although some degree of morphological polarity was present in the disk, the chlorophyll fluorescence analysis results showed that PSII photosynthetic capabilities within the disk were similar for all parts of the disk. When the tetraspores developed into young germlings, the PSII activities varied among the different parts of the germling.

## Methods

Mature reproductive tetrasporophytes of *G. vermiculophylla *were collected from the intertidal zone of Zhan Shan, Qingdao (36°05'N, 120°18'E), China. Fronds were rinsed three times in sterilized seawater to remove epiphytes from the thallus surface.

### Release and culture of tetraspores

For spore release, the rinsed tetrasporophytes were cultivated in sterilized Petri dishes containing PES medium [35] at 20°C under a photo regime of 12 hours light: 12 hours dark with light intensity of 30 μmol m^-2 ^s^-1^. The fronds were removed as soon as the tetraspores were released in abundance. The culture conditions of the tetraspores were the as same as those of the fronds. The culture medium was renewed every 2 days.

### Cultivation of excised disks

Disks, which developed from single-cell tetraspores 2 weeks after release, were excised in situ. The remaining part adhering to the slide was cultivated continuously in the same constant culture conditions. Morphological and photosynthetic changes in the remainders were photographed and described at 24 hours intervals.

### Scanning Electronic Microscopy (SEM)

Developing disks were observed by SEM, and the samples for SEM were treated as follows: Disks were detached from the slides and fixed with 0.1 M PBS (pH 7.2) containing 5% glutaraldehyde for 120 min. The samples then were rinsed four times with 0.1 M PBS, resuspended in an ethanol gradient for dehydration, and dried with a carbon dioxide critical point drier (HPC-2) (Hitach Co., Japan). An ion coater (IB-3) (Eiko Engineering, Ibaraki, Japan) was used to sputter metals onto the samples' surface. The samples then were observed and photographed using a KYKY2800B SEM (Kyky, Beijing, China).

### Preparation of serial paraffin slices

Detached disks of various sizes were fixed in 4% formaldehyde solution for 12 hours. They then were dehydrated in 70%, 83%, and 95% ethanol for 1 hour each, followed by 100% ethanol for 30 min. Before embedding in paraffin, the samples were treated as follows: ethanol/xylene (1:1) for 1 hour, 100% xylene for 30 min, xylene/paraffin for 20 min, and 100% paraffin for 20 min. The paraffin-embedded disks were sliced at a thickness of 10 μm with a Leica 2016 microtome (Leica Instruments Ltd., Shanghai, China).

### Measurement of photosynthetic parameters

Photosynthetic capability was determined by analysis of chlorophyll fluorescence, which was measured using a microscopy-PAM apparatus (Walz GmbH, Effeltrich, Germany). Imagingwin 2.21d software was used to set the parameters and analyze the fluorescence data. Before measurement, the samples, which included single-cell tetraspores, disks, and young germlings detached from the substrate, were kept in the dark for 15 min. The dark fluorescence yield, F0, was determined under low measuring light, and then a saturation pulse was applied to assess the maximal fluorescence (Fm). The Fm yield in illuminated samples was denoted as Fm'. The maximal PSII quantum yield (Fv/Fm) was calculated using the equation: Fv/Fm = (Fm - F0)/Fm. The effective PSII quantum yield was calculated as follows: Y (II) = (Fm' - F)/Fm', where the F is real time fluorescence yield. All measurements were performed at room temperature.

### Data analysis

The data were analyzed with SPSS 13.0 (SPSS Inc., Chicago, IL). Statistical significance was tested by one-way ANOVA. All values were expressed as means ± s.d. The graphs were plotted with OriginPro 8.0 (OriginLab Corporation, Northampton, USA).

## Authors' contributions

XX carried out the experiments and drafted the manuscript. GW conceived of the study and helped to draft the manuscript. GP and SG participated in the analysis of data. PX and JZ made substantial contributions to the cultivation of materials in the experiments. All authors read and approved the final manuscript.
